# Analysis of Growth Models in Galician × Nelore Crossbred Cattle in the First Year of Life

**DOI:** 10.3390/ani14243698

**Published:** 2024-12-21

**Authors:** Antonio Iglesias, Fernando Mata, Joaquim Lima Cerqueira, Alicja Kowalczyk, Jesús Cantalapiedra, José Ferreiro, José Araújo

**Affiliations:** 1 Department of Anatomy, Animal Production and Veterinary Clinical Sciences, Instituto de Biodiversidade Agraria e Desenvolvemento Rural, University of Santiago de Compostela, 27002 Lugo, Spain; antonio.iglesias@usc.es; 2 CISAS—Center for Research in Agrifood Systems and Sustainability, Instituto Politécnico de Viana do Castelo, Rua da Escola Industrial e Comercial Nun’Alvares 34, 4900-347 Viana do Castelo, Portugal; cerqueira@esa.ipvc.pt (J.L.C.);; 3 Escola Superior Agrária, Instituto Politécnico de Viana do Castelo, Refóios do Lima, 4990-706 Ponte de Lima, Portugal; 4 Veterinary and Animal Research Centre (CECAV), University of Trás-os-Montes and Alto Douro, Quinta de Prados, Apartado 1013, 5001-801 Vila Real, Portugal; 5 Department of Environmental Hygiene and Animal Welfare, Wrocław University of Environmental and Life Sciences, Chełmońskiego 38c, 51-630 Wrocław, Poland; alicja.kowalczyk@upwr.edu.pl; 6 Regional Ministry for the Rural Environment, Xunta de Galicia, Edificio Administrativo de Lugo, 27002 Lugo, Spain; tsukijizo@gmail.com (J.C.);; 7 CIMO—Mountain Research Centre, Instituto Politécnico de Viana do Castelo, Rua da Escola Industrial e Comercial Nun’Alvares 34, 4900-347 Viana do Castelo, Portugal

**Keywords:** Brazil, calf, crossbreeding, Galician Blond, growth curves, Nelore, veal, vitelão, zebu-type cattle

## Abstract

The veal niche market is gaining momentum in Brazil. Locally known as ‘Vitelão’, veal refers to the meat from calves slaughtered up to 12 months of age. In this study, we assessed the Galician Blond × Nelore cross as a candidate to produce veal. The aim of this study was to establish criteria for selecting 12-month-old calves suitable for slaughter. To find the best fit, we adjusted various growth models for calves up to 12 months of age. Once the best fit was determined, the selected growth model was then used to calculate the relative and instantaneous growth rates to evaluate the slaughtering potential at 12 months. Our study reveals that, under present conditions, the Logistic model is the best fit for characterizing and functionally analyzing growth from birth to 12 months of age in Galician Blond crosses with Nelore. Calves resulting from this cross experience rapid growth in their first 12 months of life, making them an excellent choice for producing high-quality veal while maintaining rusticity and adaptability to extreme environments. The results of this study could contribute to enhancing the growth management systems of Galician Blond and Nelore crosses in Brazilian grazing production systems. Additionally, they can be incorporated into genetic improvement programs as a tool for selecting animals with greater precocious growth without altering adult weight.

## Simple Summary

The veal market is picking up speed in Brazil, where it is called ‘Vitelão’. This term refers to meat from calves that are under a year old. We investigated the Galician Blond crossed with Nelore cattle to see if they could be a good choice for veal production. Our goal was to help farmers make better decisions when choosing calves for meat. We tested different growth models to find the best way to track how calves grow until they are 12 months old. We discovered that the Logistic model worked best for understanding their growth. These calves grow quickly in their first year, making them ideal for producing high-quality veal. They are also tough and can adapt well to challenging environments. Our findings can help improve the ways in which farmers manage cattle and support breeding programs that aim for faster growth without affecting their adult size.

## 1. Introduction

Zebu-type cattle (*Bos taurus indicus*) have long been renowned for their rusticity and adaptation to arid and semi-arid production systems. These cattle originated in the Indian sub-continent, have been utilized in Arabic countries, where some local breeds can still be found [1], and from there were introduced to Africa. Here, they were crossed with *Bos taurus taurus* and demonstrated remarkable adaptability, particularly in North Africa and the sub-Saharan Sahel [2].

Zebu-type cattle were later introduced to the Americas, where they proved to adapt better than European-type cattle in many arid, semi-arid, and tropical production systems. In Brazil, particularly in the nineteenth century, they gained prominence, and their reputation for adaptability led to them becoming the primary breed for beef production at the beginning of the 21st century [3]. Today, Nelore (Nel) is the predominant breed of beef cattle used in Brazil, and it adapts exceptionally well to ‘Cerrado’, the Brazilian Savanna, and ‘Caatinga’, the Northeastern shrub drylands of Brazil [4]. Nelore derives from the word ‘Ongole’, which is the original zebuine cattle brought to Brazil, giving rise to the Brazilian breed Nelore [5].

In connection with the aim to achieve higher-quality carcasses and meat cut yields, there has been a growing trend of introducing crosses with European breeds [6]. Crossbreeding is an essential technique for enhancing growth, meat quality, and adaptability in beef production systems in tropical regions [7], as well as in arid and semi-arid climates [8]. It is also a common strategy used to improve beef production systems in these regions [9].

The Galician Blond (GB) breed exhibits rapid growth [10] and matures later (with late fat deposition) [11]. GB calf meat is highly regarded not only in Galicia, Spain from where the breed originated, but also beyond its borders. It is produced from calves slaughtered up to 10 months of age and holds Protected Geographic Indication status [12]. The Galician Blond × Nelore crossbreeding program was initiated by the Association of Breeders of the Galician Blond breed (ACRUGA). It is based on the use of GB breed sires in industrial crossbreeding programs with Nel dams [13].

In Brazil, beef producers have now shifted their focus from quantity to quality and productivity in their production systems. With these aims and to address environmental concerns related to deforestation, veal meat, obtained from steers up to 12 months old, has been on the rise [14]. The crossbreeding of GB with Nel (GBN) aims to combine the rustic qualities of the former with the early maturity and meat quality characteristics of the latter [15]. In crossbreeding programs, the objectives pursued include capitalizing on the benefits of heterosis and complementarity between breeds [16]. In commercial beef crossbreeding programs, these objectives are achieved by leveraging both additive (breed differences) and non-additive (heterosis) effects [17]. A longer genetic distance between an individual’s ancestors tends to increase the individual’s fitness; therefore, its maximizing enhances the probability of heterosis [18], which explains the advantageous use of taurine breeds to sire zebu breed dams.

In studies involving the growth and development of animal species, nonlinear models have shown suitability. Nonlinear models can accurately describe the growth patterns of animals, and their parameters often have biologically meaningful interpretations [19]. It has long been recognized that animal growth can be predicted by growth curves that describe the relationship between weight and age [20]. These curves have been employed in various farm animal species to date (e.g., [21,22,23]). Different curves can be adjusted to suit different production systems and genders [21].

Thus, the first objective of this study was to fit various models to the growth of GBN cattle up to 12 months of age to determine the best fit. After the identification of the best-fit model, we used it to calculate the relative and instantaneous growth rates across the growth curve, addressing the second objective: evaluating the feasibility of slaughtering at 12 months of age. Ultimately, we aimed to provide a tool for decision-making in the selection of dams and sires to produce 12-month-old calves ready for slaughter for the ‘Vitelão’ niche market.

## 2. Materials and Methods

The study protocol used in this study was retrospectively approved by the CEEA/OH ‘Comité Ético de Experimentación Animal’ (Committee Responsible for Animal Welfare in Research) of the Complejo Hospitalario de Orense (CHOU), Spain (protocol code 002 and date of approval 27 February 2023).

### 2.1. Data

Data were collected from GBN calves raised at the Mosquera and Grandal cattle ranch located in Burí, São Paulo State in the ‘Cerrado’ of the southeast of Brazil. The ‘Cerrado’ is characterised by dry (May to September) and wet (October to April) seasons, corresponding to a savanna (*sensu lato*). The mean annual temperature is 22 °C and the rainfall averages 1500 mm annually. If the temperatures are maintained throughout the year, the same is not the case with the rain with a shortfall in the winter (June to September) [24].

The animals used in the study were born in May, were initially kept as suckling calves, and were weaned at 7 months of age. During their growth, they received the following supplementary feeding: For 209 days, a pre-starter ration was provided at a rate of 700 g per animal per day. This ration consisted of 18% crude protein (CP), 2.5% ether extract (EE), 7% fibre (F), 13% moisture (H), and 10% ash; after 150 days of starting the previous supplementary feeding, for an additional 145 days, they were supplemented with 2600 g per animal per day of a ration containing 40% CP, 2% EE, 15% F, and 18% H. During the last 80 days of the study, they also received 18 kg of corn silage and 3700 g of corn flour per animal per day. The data collected consisted of a series of monthly weights from birth to 12 months of age, including a total of *n* = 27 female calves and *n* = 20 intact male calves. The calves were weighted in a weighing platform Gradil De Madeira GP2800 Pesagem Bovina, with an LD 1050 display. The calves were weighed at 09:00 a.m. local time, and no feeding restrictions were applied before weighing, and therefore any possible weighting bias based on gut filling was minimized. The growth curves were adjusted based on the average weights of intact males and females at various ages (in days).

### 2.2. Growth Functions Studied

We used the most commonly used nonlinear functions to fit the growth data in cattle [25], and particularly Nelore cattle [26], which included the Brody [27], Logistic [28], Gompertz [29], von Bertalanffy [30], Logistic with a constant term, Gompertz with a constant term, von Bertalanffy with a constant term, and Richards [31] models. The common parameterization of these models is provided in Table 1. All these curves can be derived by modifying the parameter ‘d’ in the Richards equation [32] and expressing the equation as

*W*(*t*) = (*a*^1−*m*^ − *b* exp(−*ct*)^1−1−*m*^) (1)

where each parameter holds the following biological interpretation: ‘*a*’ represents mature body weight, ‘*b*’ is the constant of integration without significant biological interpretation, ‘*c*’ signifies the maturity rate, and ‘*m*’ is the inflection parameter [33]. The maturity rate reflects early maturity and represents the rate at which an organism approaches its mature weight [34].

### 2.3. Statistical Procedure

The parameters were estimated using both the least squares method and the Levenberg–Marquardt algorithm [35]. The curves were fitted to the data using the NLR (Nonlinear Regression) procedure within the IBM Corp.^®^ SPSS^®^ Statistics statistical package, located in Armonk, NY, USA. The version used was 28.0.2.0 (20). The best fit was determined based on several criteria, including the coefficient of determination (r^2^), residual mean squares (RMSs), and Mallow’s Cp statistic. Additionally, the following prerequisites, as outlined by Frost [36], were assessed and considered in the selection of the best-fit model:

1. The regression model exhibits linearity in the residuals, as guaranteed by the Q-Q plots.
2. The error term possesses a population mean of zero, which was confirmed through a one-sample *t*-test.
3. The independent variable ‘time’ demonstrates no correlation with the residuals, verified using Spearman’s correlation test.
4. The residuals do not exhibit autocorrelation, as assessed through the randomness of an ordered residual plot.
5. The residuals display constant variance, indicating the absence of heteroscedasticity. This assessment was made via residuals versus predicted values plot.
6. The independent variables are not correlated, which is ensured by the presence of a single independent variable, ‘time’, across all models.
7. The residuals exhibit a normal distribution, determined through a standardized residuals Q-Q plot, with mean rank assigned to ties and using Blom’s fractional rank estimation method.
8. The plots in Appendix A were used to check these prerequisites.

## 3. Results

Among the models studied, only the Richards model failed to converge for both males and females. All the other models successfully fit the data for males. In the case of the female dataset, the von Bertalanffy and Logistic models with a constant converged but failed to meet the prerequisite of a lack of correlation between the residuals and the independent variable ‘time’. Consequently, these two models were not further explored for females. Parameter estimates for intact males can be found in Table 2, while Table 3 contains the parameter estimates for females. Table 4 and Table 5 present the goodness-of-fit statistics for males and females, including the residual mean square (RMS), coefficient of determination (r^2^), and Mallow’s statistic (Cp).

Prerequisites 1 and 6 were verified directly, as explained in the previous section. Prerequisites 2 and 3 are also checked in Table 3 and Table 4. Prerequisites 4, 5, and 7 were assessed using the plots provided in Appendix A. Both the Logistic and Gompertz models fit the data well for both intact males and females. However, for females, the Cp statistic was smaller for the Logistic model. Therefore, among the growth models studied, the Logistic model provides the best fit for both intact males and females. Consequently, we chose to use the Logistic function to model both the male and female data. Utilizing the estimated parameters, the growth functions take the following form:

For intact males:

(2) $$W\left(t\right)=504.65(1+8.426\exp{\left(-0.0105t\right)}^{-1}).$$

For females:

(3) $$W\left(t\right)=432.14(1+7.630\exp{\left(-0.00957t\right)}^{-1}),$$

where W is the weight at day t.

**Figure 1:**
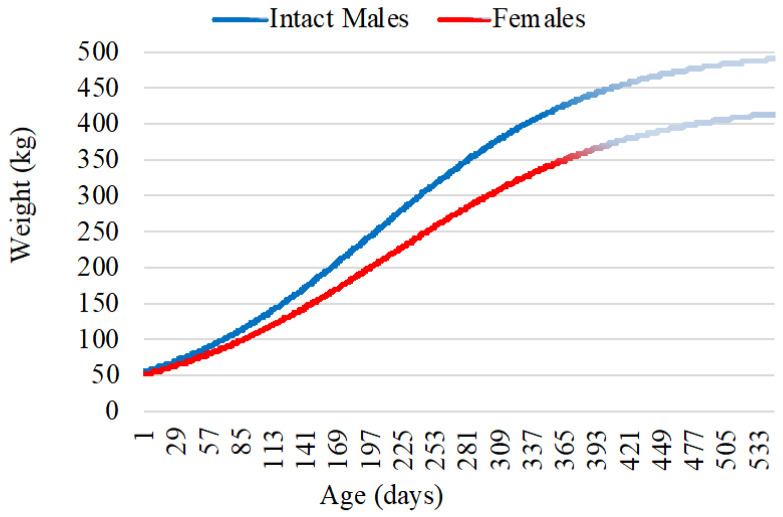
Growth curves for intact male and female crosses between Galician Blond and Nelore. Growth projection after 12 months and up to 18 months is represented in lighter colors.

Figure 1 represents the growth curves for intact males and females after the Logistic model.

The Logistic function expresses the rate of growth multiplied by a factor representing the deficit in size still to be gained,

(4) $$\left[W_{\infty }-W\left(t\right)W_{\infty }\right]$$

The differential equation can be written as

(5) $$\frac{dW\left(t\right)}{dt}=rW\left(t\right)\left[\frac{W_{\infty }-W\left(t\right)}{W_{\infty }}\right]\Leftrightarrow$$

(6) $$\Leftrightarrow W\left(t\right)=\frac{W_{\infty }}{\left[1+\left(\left(\frac{W_{\infty }}{W\left(t\right)}\right)-1\right)\exp\left(-r*t\right)\right]}\Leftrightarrow$$

With *W*_∞_, final weight = *a*, (*W*_∞_ *W*(t)) − 1 = *b*, and *r* = *c* to find the parameterization being used in the present study.

(7) $$\Leftrightarrow W\left(t\right)=\frac{a}{\left[1+b\exp\left(-ct\right)\right]}$$

**Figure 2:**
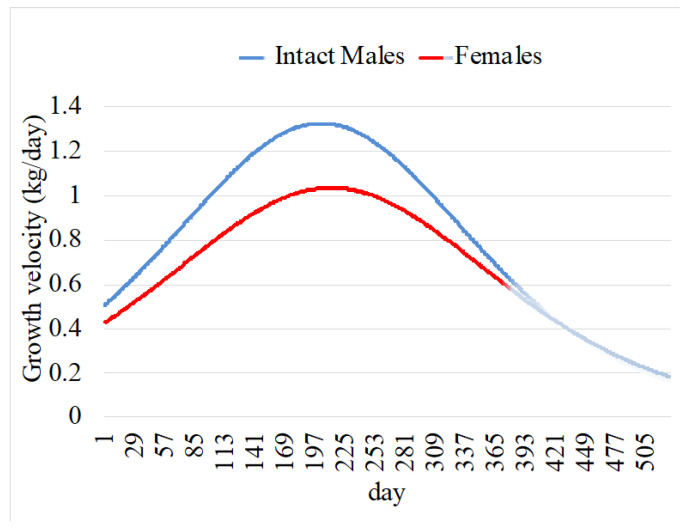
Growth velocity or relative growth rate over time for Galician Blond × Nelore crosses. The projection of growth after 12 months of age and up to 18 months is represented in lighter colors.

**Figure 3:**
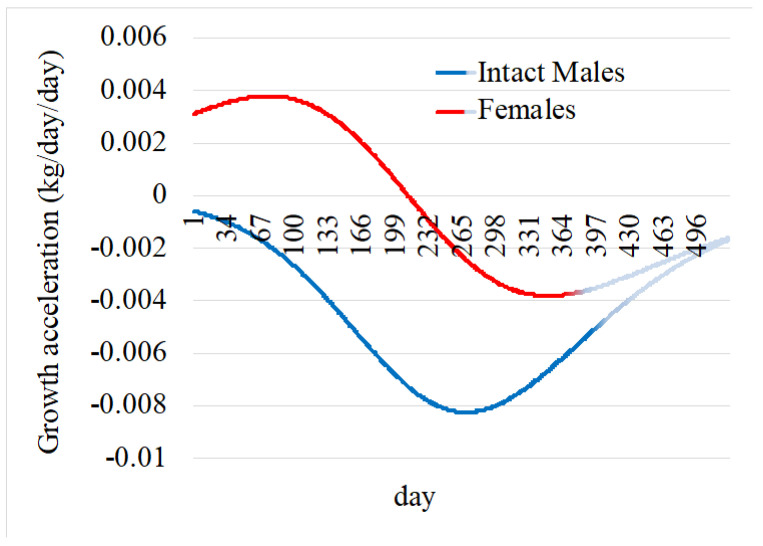
Growth acceleration or instantaneous growth rate over time for Galician Blond × Nelore crosses. The projection of growth after 12 months of age and up to 18 months is represented in lighter colors.

The first derivative functions (Equation (8) for intact males and Equation (9) for females) can be used to represent the relative growth rate through time (growth velocity) (Figure 2), and the second derivative functions (Equation (10) for intact males and Equation (11) for females) represents the instantaneous growth rate through time (growth acceleration) (Figure 3).

(8) $$\frac{dW\left(t\right)}{dt}\left(males\right)=\frac{892957989*\exp\left(\frac{21t}{2000}\right)}{80\cdot {\left(500\cdot \exp\left(\frac{21t}{2000}\right)+4213\right)}^{2}}$$

(9) $$\frac{dW\left(t\right)}{dt}\left(females\right)=\frac{15777236937*\exp\left(\frac{957t}{100000}\right)}{5000\cdot {\left(100\cdot \exp\left(\frac{957t}{100000}\right)+763\right)}^{2}}$$

(10) $$\frac{d^{2}W\left(t\right)}{dt}\left(males\right)=-\left(\frac{18752117769\cdot (500*\exp\left(\frac{21t}{1000}\right)-4213\cdot \exp\left(\frac{21t}{2000})\right)}{{16000\cdot \left(500\cdot \exp\left(\frac{21t}{2000}\right)+4213\right)}^{3}}\right)$$

(11) $$\frac{d^{2}W\left(t\right)}{dt}\left(females\right)=-\left(\frac{15098815748709\cdot (100\cdot \exp\left(\frac{957t}{50000}\right)-763\cdot \exp\left(\frac{957t}{100000})\right)}{5000000000\cdot (100\cdot \exp\left(\frac{957t}{100000}\right)+{763)}^{3}}\right)$$

## 4. Discussion

Various growth models have been applied to study cattle growth. According to Forni et al. [37], traditional models such as Brody, Gompertz, von Bertalanffy, and Logistic are suitable for establishing mean growth patterns and predicting adult body weight. However, the Brody model stands out for its simplicity and accuracy in predicting birth weight, even though, in our study, the Logistic function provided the best fit, albeit with an overestimated birth weight. Other researchers like Freitas [38] have also observed that the Logistic function tends to estimate higher birth weights.

In a comprehensive review by Freitas [38], the Logistic and Von Bertalanffy models emerged as the most versatile for fitting growth data across eight different species, including beef cattle. However, in our study, the Richards function was the only one that failed to converge, aligning with warnings about its convergence challenges from researchers such as Crispim et al. [34].

In our study, we look into models fitting the first year of growth, and the curve patterns differ from those when animals are slaughtered. The logistic curve proves to be the best fit with very high degrees of adjustment. Its parameters could therefore serve in breeding programs for progeny selection having as aim the production of ‘Vitelão’, and despite overestimating birth weight. Different calves have different genetics, and therefore growing patterns. As growth evolves, the prediction of final weight becomes more accurate. Growth and weight in cattle are dependent on genetic and environmental factors; therefore, birth weight cannot accurately estimate slaughtering weight [39,40]. Analyzing the growth, growth rate, and instantaneous growth rate curves reveals that GBN calves exhibit the precocity to be slaughtered at 12 months of age. The growth rate is rapid up to this age, with growth patterns at 12 months reaching minimum instantaneous growth and daily gains approaching lower levels. These observations are more pronounced in intact males than in females, as expected.

The growth patterns of gender differ due to hormonal, metabolic, and physiological factors [41]. Intact males also exhibit superior feed efficiency, converting feed into muscle more effectively [42]. Intact males grow faster, larger, and for a longer duration due to testosterone, which promotes muscle accretion and delays fat deposition, leading to a higher lean-to-fat ratio. Females, on the other hand, exhibit earlier fat deposition due to estrogen production, reducing growth velocity and limiting overall size [43,44]. Growth velocity peaks later intact males and is sustained longer, whereas in females, it peaks earlier and declines with fat deposition as they mature [45]. Growth acceleration, the change in growth velocity, is greater and prolonged in intact males due to their later maturity, and this was previously reported for Nelore cattle [46].

The predicted weight at 12 months stands at 427 kg for males and 351 kg for females, consistent with the results obtained for intact males by Sánchez et al. [10] (447 kg). It is noteworthy that GBN calves exhibit superior growth patterns, namely speed, and muscular mass, up to 12 months compared to crosses between Nelore and other specialized beef breeds. The GB breed carries mutations of the myostatin double-muscled gene (MSTN) [47]. The MSTN mutation impairs the regulator function of muscle growth, leading to double muscling [48]. Typically, breeds carrying the MSTN gene have an increased muscle mass and a decreased fat deposition in the carcass [49]. This leads to leaner meat, improved feed efficiency, increased carcass value with a higher proportion of noble cuts, and higher profitability for producers [49]. It also offers genetic advantages for selective breeding [50]. A crossbred calf such as the GBN therefore shows increased muscular mass [51]. This results in increased meat tenderness due to lower collagen content and a higher number of muscular fibres in double-muscled cattle [52].

The GB is homozygous for the MSTN gene. Therefore, all the animals of the breed have double muscling. The GBN calves are heterozygous for the MSTN gene. However, a strategy for enhancing the heterozygosity of the MSTN allele in beef cattle while minimizing homozygosity results in leaner, more muscular carcasses [53].

Crossbreeding *Bos indicus* with *Bos taurus* also improves the tenderness of the traditionally less tender *Bos indicus* meat. Zebu-type cattle show a higher activity of the calpain-calpastatin enzyme complex, with a higher level of calpastatin activity in zebu-type cattle, reducing post mortem proteolysis and negatively affecting the tenderness of the meat [54,55]. This improved tenderness also leads to larger carcasses and cut yields [56]. Despite being later maturing breeds, GB and GBN compensate for the lack of fat with tenderness and organoleptic properties [51], thereby increasing the protein content in the meat. This makes GBN calf meat a healthier choice compared to other red meats [55], presenting the potential for branding by the meat industry.

Another advantage of this meat is its lower cooking losses compared to other veal. According to [51], the meat of double-muscled animals with low-fat deposition retains a higher water content. Nevertheless, crossbreeding double-muscled European cattle with zebu-type cattle also presents some challenges, like unpredictable offspring performance, increased dystocia, poorer heat tolerance, and inconsistent growth rates [57].

## 5. Conclusions

GBN calves exhibit rapid growth in their first 12 months, making them an excellent choice for producing high-quality veal while maintaining rusticity and adaptability to challenging environments such as the Brazilian ‘Cerrado’. Under the conditions of our study, the Logistic model is a suitable choice for characterizing and functionally analyzing growth from birth to 12 months of age in GBN. The findings of this study can contribute to improving growth management systems for GBN in grazing production systems in the Brazilian ‘Cerrado’ and inform genetic improvement programs, assisting in the selection of animals with greater precocious growth without altering adult weight, a feature not achievable when selecting solely based on weight at a specific age. The breeder may use the biological interpretation of the logistic model to aid in the selection of sires through mature body weight and maturity rate.

## Figures and Tables

**Table 1:** Equations of the different functions used to fit the growth data of the Galician Blond × Nelore crossed calves. All the functions but the Richards were fitted with and without an additional constant.

Model	Equation
Brody	$W\left(t\right)=a(1-b\exp\left(-ct\right))$
Gompertz	$W\left(t\right)=a(\exp(-b\exp\left(-ct\right))$
Logistic	$W\left(t\right)=a(1+b\exp({-ct))}^{-1}$
von Bertalanffy	$W\left(t\right)=a(1-b\exp({-ct))}^{3}$
Richards	$W\left(t\right)=a(1-b\exp({-ct))}^{d}$

Note: *W*(*t*)—weight at time *t*; *a*, *b*, and *d* are parameters of the equations.

**Table 2:** Parameters and confidence intervals of the adjusted equations (intact males).

Functions for Males	Parameter			
	a	b	c	Constant (C)
Brody	1851.0	0.997	7.2 × 10^−4^	
Logistic	504.65	8.426	0.0105	
Gompertz	595.32	2.747	5.81 × 10^−3^	
von Bertalanffy 2/3	26.225	5.642	4.24 × 10^−3^	
Brody + C	305.42	6.045	7.2 × 10^−4^	1546.05
Logistic + C	638.55	3.778	7.34 × 10^−3^	−113.91
Gompertz + C	635.29	2.487	5.4 × 10^−3^	−0.922
von Bertalanffy 2/3 + C	25.896	5.811	4.42 × 10^−3^	10.936
Richards	Did not converge			

**Table 3:** Parameters and confidence intervals of the adjusted equations (females).

Functions for Females	Parameter			
	a	b	c	Constant (C)
Brody	16,843	0.999	5.54 × 10^−5^	
Logistic	432.14	7.630	957 × 10^−3^	
Gompertz	561.14	2.610	4.75 × 10^−3^	
von Bertalanffy 2/3	237.02	2.0000	5.85 × 10^−3^	
Brody + C	6716.49	1.615	8.64 × 10^−5^	4152.78
Logistic + C	266.60	−0.096	−7.06 × 10^−7^	−240.99
Gompertz + C	638	2.310	4.12 × 10^−3^	−26.913
von Bertalanffy 2/3 + C	705.77	5.462	5.06 × 10^−3^	−5.015
Richards	Did not converge			

**Table 4:** Indicators of the quality of adjustment of the different growth models (intact males).

Male Model	RMS	r^2^	Cp	$E\bar{x}$	ρEt
Brody	557	0.96	1.03	0 ^NS^	0.011 ^NS^
Logistic	545	0.96	1.02	0 ^NS^	0.008 ^NS^
Gompertz	521	0.96	1.02	0 ^NS^	−0.022 ^NS^
von Bertalanffy 2/3	690	0.96	1.02	0 ^NS^	−0.022 ^NS^
Logistic + C	536	0.96	2.00	0 ^NS^	−0.031 ^NS^
Gompertz + C	534	0.96	1.99	0 ^NS^	−0.012 ^NS^
von Bertalanffy 2/3 + C	533	0.96	2.02	0 ^NS^	−0.020 ^NS^
Richards	Did not converge				

RMS—residual mean square, r^2^—coefficient of determination, Cp—Mallow’s Cp, E$\bar{x}$—error term mean of zero, confirmed through a one-sample *t*-test, ρEt—correlation of the variable ‘time’ with the residuals after the Spearman’s correlation test. NS—non-significant.

**Table 5:** Indicators of the quality of adjustment of the different growth models (females).

Female Model	RMS	r^2^	Cp	$E\bar{x}$	ρEt
Brody	794	0.91	3.99	0 ^NS^	0.057 ^NS^
Logistic	791	0.91	3.99	0 ^NS^	0.065 ^NS^
Gompertz	787	0.91	4.00	0 ^NS^	0.074 ^NS^
von Bertalanffy 2/3	5706	0.35	4.00	0 ^NS^	0.866 ^NS^
Brody + C	814	0.91	4.98	0 ^NS^	0.060 ^NS^
Logistic + C	1826	0.80	5.00	0 ^NS^	0.506 ^NS^
Gompertz + C	805	0.91	5.02	0 ^NS^	0.070 ^NS^
von Bertalanffy 2/3 + C	806	0.91	5.00	0 ^NS^	0.068 ^NS^
Richards	Did not converge				

RMS—Residual mean square, r^2^—Coefficient of determination, Cp—Mallow’s Cp, E$\bar{x}$—error term mean of zero, confirmed through a one-sample *t*-test, ρEt—correlation of the variable ‘time’ with the residuals after the Spearman’s correlation test. NS—non-significant.

**Figure A1:**
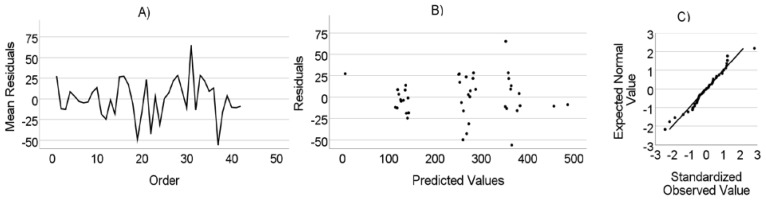
Brody model for males. (**A**) Ordered residual plot, (**B**) residuals versus predicted value plot, (**C**) standardized residual Q-Q plot.

**Figure A2:**
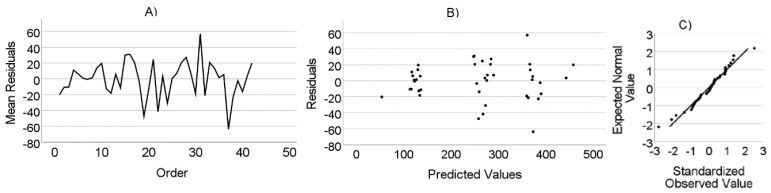
Logistic model for males. (**A**) Ordered residual plot, (**B**) residuals versus predicted value plot, (**C**) standardized residual Q-Q plot.

**Figure A3:**
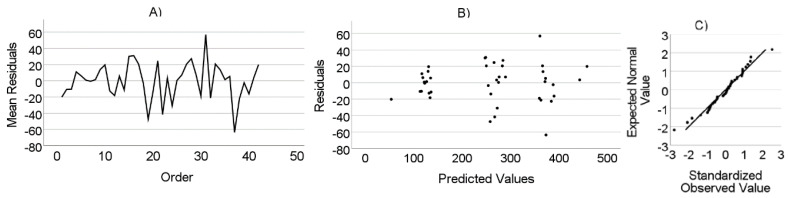
Gompertz model for males. (**A**) Ordered residual plot, (**B**) residuals versus predicted value plot, (**C**) standardized residual Q-Q plot.

**Figure A4:**
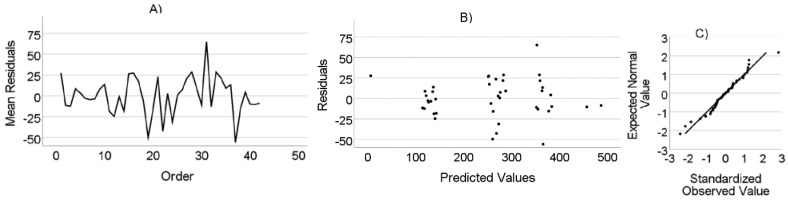
Von Bertalanffy 2/3 model for males. (**A**) Ordered residual plot, (**B**) residuals versus predicted value plot, (**C**) standardized residual Q-Q plot.

**Figure A5:**
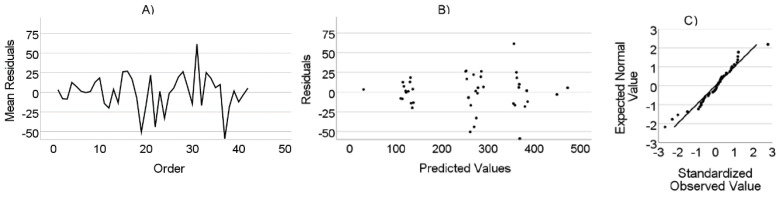
Brody + constant model for males. (**A**) Ordered residual plot, (**B**) residuals versus predicted value plot, (**C**) standardized residual Q-Q plot.

**Figure A6:**
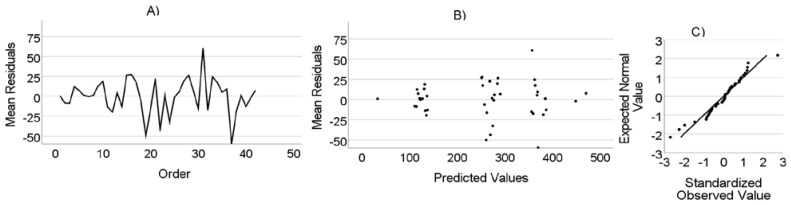
Logistic + constant model for males. (**A**) Ordered residual plot, (**B**) residuals versus predicted value plot, (**C**) standardized residual Q-Q plot.

**Figure A7:**
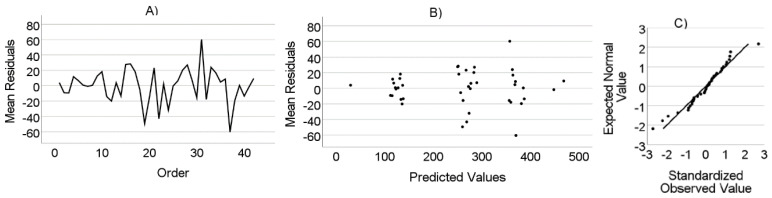
Gompertz + constant model for males. (**A**) Ordered residual plot, (**B**) residuals versus predicted value plot, (**C**) standardized residual Q-Q plot.

**Figure A8:**
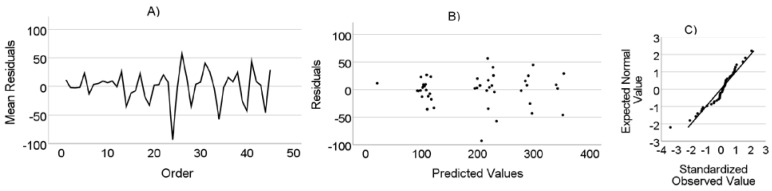
Bertalanffy 2/3 + constant model for males. (**A**) Ordered residual plot, (**B**) residuals versus predicted value plot, (**C**) standardized residual Q-Q plot.

**Figure A9:**
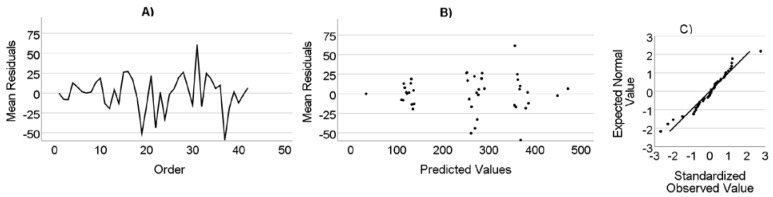
Brody model for females. (**A**) Ordered residual plot, (**B**) residuals versus predicted value plot, (**C**) standardized residual Q-Q plot.

**Figure A10:**
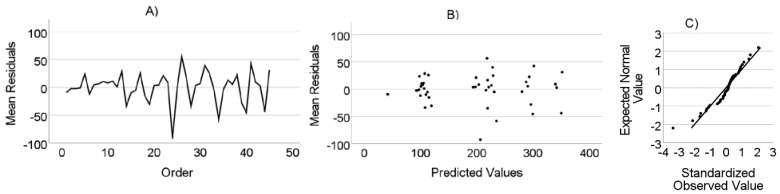
Logistic model for females. (**A**) Ordered residual plot, (**B**) residuals versus predicted value plot, (**C**) standardized residual Q-Q plot.

**Figure A11:**
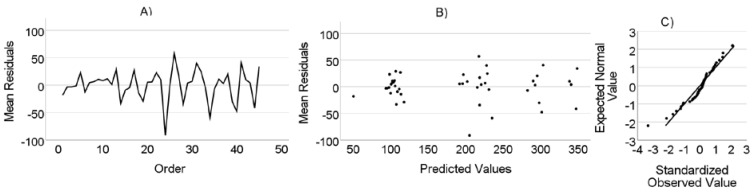
Gompertz model for females. (**A**) Ordered residual plot, (**B**) residuals versus predicted value plot, (**C**) standardized residual Q-Q plot.

**Figure A12:**
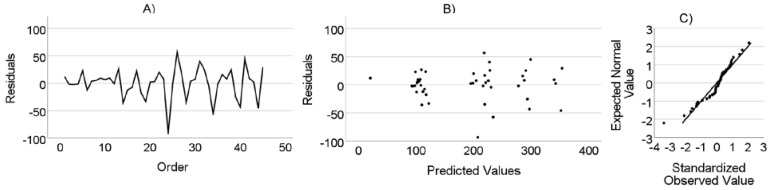
Brody + constant model for females. (**A**) Ordered residual plot, (**B**) residuals versus predicted value plot, (**C**) standardized residual Q-Q plot.

**Figure A13:**
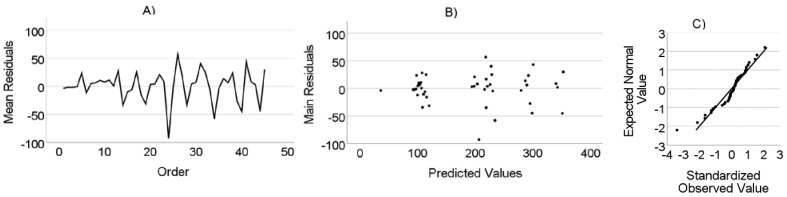
Gompertz + constant model for females. (**A**) Ordered residual plot, (**B**) residuals versus predicted value plot, (**C**) standardized residual Q-Q plot.

**Figure A14:**
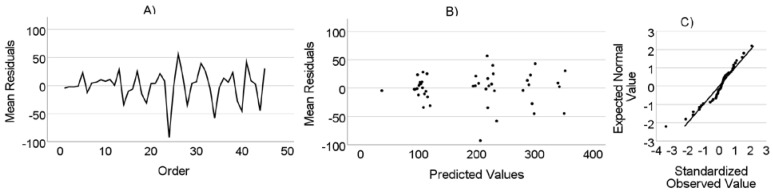
Bertalanffy + constant model for females. (A) Ordered residual plot, (**B**) residuals versus predicted value plot, (**C**) standardized residual Q-Q plot.

## Data Availability

The raw data supporting the conclusions of this article will be made available by the authors upon request.
